# Recent advances in liver disease

**Published:** 2016

**Authors:** David Al Dulaimi

**Affiliations:** *Department of Gastroenterology, Alexandra Hospital, Redditch, UK*

*Mohammadali F, Pourfathollah AA* *.* ***Changes in frequency of HBV, HCV, HIV and syphilis infections among blood donors in Tehran province 2005 - 2011.*** *Arch Iran Med 2014; 17(9)613-620*

The routine screening of blood donors for potentially transmissible infections has reduced transfusion-associated infection whilst providing an insight into disease prevalence. This retrospective cross-sectional study evaluates the prevalence of hepatitis B virus (HBV), hepatitis C virus (HCV), human immunodeficiency (HIV) and syphilis infection in 2,026,628 potential blood donors screened at Tehran Blood Transfusion Centre from 2005 to 2011. One or more transmissible infections were identified in 0.52% of donors (mean age 38±10.5 y, 95% male) with greater prevalence in first time donors. The average prevalence rate of HBV, HCV, syphilis and HIV was 388, 112, 10.5 and 5.4 per 100,000 respectively. A decreasing prevalence rate of HBV was identified from 2005 to 2011 and of HCV from 2007 to 2011. This may have resulted from increased vaccination, screening and health education. Although an interesting insight into disease prevalence in the donor population, caution should be exercised in extrapolating these results to the wider population in view of sampling bias.

*De BK et al.* ***Pentoxifylline plus prednisolone versus pentoxifylline only for severe alcoholic hepatitis: a randomised controlled clinical trial.*** *Ann Med Health Sci Res 2014;4(5):810-816*

Pentoxifylline and prednisolone are individually useful in the management of severe acute alcoholic hepatitis (Maddrey discriminant function (MDF) ≥32) but their combined efficacy is unclear. This single-centre randomised controlled trial investigated the short and medium term outcomes of treatment with pentoxifylline and prednisolone compared to pentoxifylline alone in 60 patients with severe alcoholic hepatitis from 2010 to 2012. During the 12 month follow up period 33% and 20% of patients died in the dual therapy and monotherapy groups respectively. Overall there was no significant difference in mortality and morbidity between the treatment groups. The study could have been enhanced by including a third prednisolone monotherapy group for comparison.

*Iwaisako K et al.* ***Origin of myofibroblasts in the fibrotic liver in mice.*** *Proc Natl Acad Sci USA 2014;111(32):E3297-3305.*

Activated hepatic myofibroblasts are responsible for fibrous scar formation in chronic liver disease although their origin is unclear. Using transgenic reporter mice this study investigates the origin of myofibroblasts and quantifies their number in hepatocellular and cholestatic-type chronic liver disease induced by carbon tetrachloride (CCl_4_) administration and common bile duct ligation (BDL) respectively. Myofibroblast activation and the degree of hepatic fibrosis was similar in both types of liver injury. In CCL_4_- or alcohol-induced fibrosis, activated hepatic stellate cells (aHSCs) comprised >92% of myofibroblasts whereas in BDL-induced liver injury myofibroblasts originated from activated portal fibroblasts (aPFs) and aHSCs in similar proportions. Of note, the genotype of BDL-aHSCs was more similar to aPFs than CCL4-aHSCs suggesting that following cholestatic liver injury aHSCs may mimic aPFs. In BDL-induced liver injury PF activation may lead to cytokine production causing subsequent HSC activation. The observation that mesothelin expression is unregulated in aPFs following BDL injury may offer a novel target for future anti-fibrotic therapy. Hepatotoxic and cholestatic liver injuries activate distinct subsets of fibrogenic myofibroblasts, preferentially aHSCs and aPFs respectively. 

*Nassar Junior AO, et al.* ***Terlipressin versus norepinephrine in the treatment of hepatorenal syndrome: a systemic review and meta-analysis.*** *PLoS One 2014;9(9):e107466*

Hepatorenal syndrome (HRS) is a severe, progressive renal failure associated with end-stage liver disease which may require splanchnic vasoconstrictor therapy whilst liver transplantation is awaited. In some countries the cost and poor availability of terlipressin has prompted search for an alternative splanchnic vasoconstrictor with similar efficacy. This systematic review and meta-analysis of four studies comprising 154 adult patients compared the efficacy and safety of norepinephrine with terlipressin in the management of HRS. Both HRS type 1 and 2 was analysed and all patients received intravenous albumin and central venous pressure monitoring. There was no difference in the reversal of HRS (RR 0.97, 95% CI 0.76-1.23), mortality at 30 days (RR 0.89, 95% CI 0.68-1.17) and recurrence of HRS (RR 0.72, 95% CI 0.36-1.45) between the norepinephrine and terlipressin treatment groups. Adverse events were less common with norepinephrine (RR 0.36, 95% CI 0.15-0.83) although all were of minor importance. Although norepinephrine appears an attractive alternative to terlipressin with favourable cost and availability in some countries, the authors highlight significant bias. A large randomised controlled trial is needed to further evaluate splanchnic vasoconstrictor therapy in HRS. 

*Johal AS, et al* *.* ***Endoscopic ultrasound-guided liver biopsy in pediatric patients.*** *Endosc Ultrasound 2014:3(3)191-194*

Endoscopic ultrasound-guided liver biopsy (EUS-LB) requires further evaluation in the pediatric population. This case series reports EUS-LB using a 19-gauge aspiration needle in three pediatric patients aged 9, 14 and 17 years for the evaluation of deranged liver enzymes. All patients were sedated with propofol. Colour doppler imaging determined a safe needle path prior to puncture. In all cases EUS-LB yielded sufficient tissue to make an accurate histopathologic diagnosis (aggregate tissue length 30-62 mm, 16-31 complete portal tracts). No immediate or delayed complications were reported. The authors conclude that EUS-LB is a safe, diagnostically useful technique in the pediatric population. This should be confirmed in further studies with larger patient cohorts. 

*Celikbilek M et al.* ***Circulating microRNAs in patient with non-alcoholic fatty liver disease.*** *World J Hepatol 2014;6(8):613-620*

There is a need for accurate minimally invasive tests to inform the diagnosis of non-alcoholic fatty liver disease (NAFLD) and reduce reliance on liver biopsy. The serological measurement of microRNAs (miRNA) is attractive but further work is needed to characterise those miRNA specific for NAFLD and whether levels correlate with disease severity. This cross-sectional study investigates the expression of several miRNAs sampled serologically in 20 adult patients with histologically proven NAFLD and 20 healthy age-matched controls between 2010 and 2012. The expression of miR-181d, miR-99a, miR-197 and miR-146b was significantly lower in patients with NAFLD compared to controls (p<0.05). Levels of miR-197 and miR-10b inversely correlated with the degree of inflammation (p<0.05) and miR-181d and miR-99a inversely correlated with serum gamma glutamyl transferase levels in NAFLD (p<0.05). The expression of miR-10b, miR-122, miR-34a and miR-29a did not differ between NAFLD and control groups. Overall this study identifies an altered serum miRNA expression pattern associated with NAFLD. By further characterising NAFLD-associated miRNAs future work should aim to develop a serological diagnostic test for NAFLD.

*Shi M* *, et al* *.* ***Statin use and risk of liver cancer: an update meta-analysis.*** *BMJ open 2014;4:e005399*

In addition to their principle cholestrol-lowering action statins may offer anti-cancer benefits. This meta-analysis of 12 studies (one IPD analysis of 22 RCTs, five cohort studies, six case control studies) investigated the effect of statins in 35,756 patients with liver cancer from 2005 to 2013. Statin use was associated with a reduced risk of liver cancer only when data from observational studies was sub-analysed (RR=0.57, p=0.03). There was a trend towards a greater liver cancer protective effect with statin use in patients at high risk of liver cancer (older age, HBV, HCV infection). Overall the observed risk reduction in liver cancer cannot be clearly attributed to a direct statin-mediated effect due to several confounding factors including inter-study variability in statin indications and contraindications and other confounding lifestyle changes which may accompany statin use. The effect of statins in patients at high risk for liver cancer requires further evaluation before statins can be considered an adjuvant treatment for liver cancer.

Other contributors to this page are **Dr Ishaq Ahmad** and **Luke Materacki**, Alexandra Hospital, UK

